# Is extracorporeal shock wave therapy more effective in younger patients in the treatment of mild-to-moderate erectile dysfunction? A prospective controlled study

**DOI:** 10.1093/sexmed/qfag069

**Published:** 2026-07-31

**Authors:** İbrahim Halil Albayrak, Mehmet Demir, Yavuz Onur Danacıoğlu, Tuncay Toprak, Alp Kavukçu, Eyyüp Sabri Pelit

**Affiliations:** Department of Urology, Harran University, 63290 Şanlıurfa, Turkey; Department of Urology, Harran University, 63290 Şanlıurfa, Turkey; Department of Urology, Medipol University, 34214 Istanbul, Turkey; Department of Urology, University of Health Sciences, Hamidiye Faculty of Medicine, FSM Health, 34668 Istanbul, Turkey; Department of Urology, Harran University, 63290 Şanlıurfa, Turkey; Department of Urology, Harran University, 63290 Şanlıurfa, Turkey

**Keywords:** international index of erectile function (IIEF), erectile dysfunction, extracorporeal shock wave therapy, age factors

## Abstract

**Introduction:**

The influence of patient age on treatment outcomes of extracorporeal shock wave therapy (ESWT) for mild-to-moderate erectile dysfunction remains uncertain. This study aimed to evaluate the efficacy of extracorporeal shock wave therapy according to the patient’s age.

**Methods:**

Patients with mild-to-moderate erectile dysfunction (ED, International Index of Erectile Function [IIEF] score 11-25) who were not receiving any medical or intracavernosal therapy were prospectively included in the study. The patients were divided into 2 age groups: <50 years and ≥ 50 years. All patients underwent the same ESWT protocol. No additional treatments were given to patients during the ESWT period or during follow-up. International Index of Erectile Function and erection hardness score (EHS) were recorded at baseline and at 1, 3, and 6 months. The effectiveness of ESWT was compared between the groups.

**Results:**

A total of 52 individuals were included in the study, with a mean age of 49.6 ± 13.6 years. There were no statistically significant differences in baseline IIEF scores and EHSs between the 2 groups (*P* > .05). In the < 50 years group, the IIEF scores at 1, 3, and 6 months post-ESWT were 21.9, 22.1, and 21.9, respectively. In the ≥ 50 years group, the corresponding scores were 13.3, 17.6, and 19, respectively. At 1, 3, and 6 months after ESWT, the IIEF scores of the group aged ≥ 50 years were significantly lower than that of the group aged < 50 years (*P* < .05). Erection hardness scores increased in both groups at 1, 3, and 6 months after ESWT; however, the magnitude of this increase was similar between the groups (*P* > .05).

**Conclusion:**

The findings of this study indicate that ESWT demonstrates higher clinical success rates in the younger patient group with mild-to-moderate ED compared with the older patient group. Extracorporeal shock wave therapy may be considered an effective and sufficient standalone treatment option in young patients with ED.

## Introduction

Erectile dysfunction (ED) is a common health problem that is being increasingly observed in young individuals and adversely affects sexual quality of life and psychosocial well-being. Although ED is often associated with aging, a notable increase has also been observed in younger populations in recent years. Lifestyle changes, stress, obesity, smoking, and the increasing prevalence of metabolic syndrome are among the principal factors driving this rise.^1^

Pharmacological approaches used in the treatment of erectile dysfunction (such as phosphodiesterase type 5 inhibitors) provide symptomatic improvement but do not address the underlying pathophysiological mechanisms and may have limited long-term efficacy.^2^ Therefore, there is a growing need for treatment modalities that target etiological mechanisms and offer the potential for durable recovery.

Extracorporeal shock wave therapy (ESWT) is a noninvasive treatment approach developed to exploit the effects of low-intensity shock waves on tissues, which promote angiogenesis, regeneration, and vascular repair. Initially, this therapy was used in the treatment of orthopedic conditions; however, in recent years, it has yielded promising results in ED management. In particular, ESWT has been reported to induce new capillary formation in the penis, improve microvasculature, and activate stem cell–mediated pathways that support neural repair.^3^

Recent studies have reported successful outcomes with ESWT in ED treatment in approximately 70%-85% of patients. However, a limited number of studies have evaluated the efficacy of ESWT specifically in younger populations, and the paucity of evidence in this area warrants further investigation.^4^

It has been suggested that the response to ESWT may be more favorable in younger patients due to better preservation of vascular structures. We addressed the following question: Can ESWT as a monotherapy provide effective and long-lasting results in young patients?

In this prospective controlled study, we compared the effects of ESWT by age in patients with mild and moderate ED, with the aim of assessing whether the patient’s age affects the therapeutic efficacy of ESWT.

## Materials and methods

Following ethics committee approval (HRÜ/24.06.25), 52 patients were enrolled prospectively between 2024 and 2025 after providing written informed consent. The patients were recruited from 2 centers, one a university hospital and the other a private clinic. A routine physical examination was performed on all patients, and their body mass index (BMI), total testosterone, prolactin, glucose, LDL, HDL, triglycerides, and HbA1c were measured. Comorbidities and the use of medications or therapies that could affect erectile function, including phosphodiesterase-5 (PDE5) inhibitors and intracavernosal injections, were recorded.

Inclusion criteria were as follows: age ≥ 18 years, at least 6 months of ED complaints, mild-to-moderate ED (International Index of Erectile Function [IIEF] 11-25), and no prior medical or intracavernosal treatment for ED. Patients were divided into 2 groups according to age: <50 years and ≥ 50 years. All patients underwent the same ESWT protocol. The patients did not take PDE5 inhibitors during the ESWT period or during follow-up.

Exclusion criteria were as follows: penile deformity or Peyronie’s disease; testicular failure; psychogenic ED; severe ED (IIEF < 10); neurological or cardiovascular disease; use of antihypertensive, antidepressant, antiepileptic or sedative medications that could affect erection; uncontrolled diabetes (HbA1c > 9); previous major pelvic surgery (radical prostatectomy, pelvic exenteration, or colorectal cancer surgery); and prior medical or intracavernosal treatments for ED.

International Index of Erectile Function scores and erection hardness scores (EHS) were recorded for all patients at the initial presentation. Follow-up scoring was performed and recorded at 1, 3, and 6 months.

### Low-intensity (LI)-ESWT procedure

Low-intensity extracorporeal shock wave therapy (LI-ESWT) was administered to patients twice weekly for 3 weeks, for a total of 6 sessions, using the Modus Focus ESWT device (Modus ED SWT, Inceler Medical, Ankara, Turkey). The system utilizes electrohydraulic technology to produce focused shock waves. Applied treatment parameters followed established LI-ESWT protocols with proven safety and clinical effectiveness for the management of erectile dysfunction. After manual retraction of the penis, shock waves were applied to 5 different points on the right and left lateral aspects of the corpora cavernosa, delivering 300 shocks to each point for a total of 1500 shock waves. The energy density was set at 0.15 mJ/mm^2^ and the frequency at 3 Hz. The same protocol was applied to all patients.

### Statistical analysis

Descriptive statistics were presented as mean, SD, median, minimum, maximum, frequency, and percentage values. Distribution of variables was assessed using the Kolmogorov–Smirnov and Shapiro–Wilk tests. The Mann–Whitney *U* test was used for the analysis of independent quantitative variables that did not follow a normal distribution. The chi-square test was used for categorical independent variables; when chi-square test assumptions were not met, Fisher’s exact test was applied. Linear mixed model and analysis of covariance (ANCOVA) were also used where appropriate. All analyses were performed using SPSS version 27.0.

## Results

A total of 52 patients were included in the study. The patients were divided into 2 groups using 50 years of age as the cutoff (<50 years and ≥ 50 years). Participants’ ages ranged from 26 to 80 years, with a mean age of 49.6 ± 13.6 years. Nineteen patients (36.5%) had comorbid conditions: 11 had diabetes mellitus, and 8 had hypertension. Demographic characteristics of the patients are summarized in Table 1.

**Table 1:** Demographic characteristics of patient population.

		Min–max	Median	Mean ± SD/*n* %		
Age		26.0–80.0	49.0	49.6 ± 13.6		
Age	<50			27		51.9%
	≥50			25		48.1%
BMI (kg/m^2^)		20.0–37.0	25.0	26.0 ± 3.7		
Smoking	(-)			34		65.4%
	(+)			18		34.6%
Comorbidity	(-)			33		63.5%
	(+)			19		36.5%
*DM*				11		57.9%
*HT*				8		42.1%
*Psoriasis*				1		5.3%
*Chronic kidney disease (CKD)*				2		10.5%
*Peynorie*				1		5.3%
*Asthma*				1		5.3%
HbA1c		5.00–11.00	6.00	6.34±1.10		
Duration of erectile dysfunction (years)		0.25–9.00	3.00	3.64±2.22		
IIEF-5 score		10.0–22.0	12.0	13.0±2.6		
EHS		0.00–3.00	1.00	1.46±0.73		
Total testosterone (ng/dL)		188.0–1362.0	388.0	438.4±191.0		

Abbreviations: BMI, body mass index; DM, diabetes mellitus; EHS, erection hardness score; HT, hypertension; IIEF, International Index of Erectile Function.

The smoking rate was higher in the < 50 years group (51.9%) than in the ≥ 50 years group (16.0%) (*P* = .007). The prevalence of comorbidities was significantly higher in the ≥ 50 years group (*P* = .026). The prevalence of diabetes mellitus was similar between groups, with 5 patients in the < 50 age group and 6 patients in the > 50 age group. There was no significant difference between the 2 groups with respect to HbA1c levels (*P* > .05). Pretreatment testosterone was significantly lower in the ≥ 50 years group compared with the < 50 years group (*P* = .010; Table 2).

**Table 2:** Comparison of groups.

		Age < 50 (*n*:27)			Age ≥ 50 (*n*:25)		*P*		
		Mean ± SD/*n* %		Median		Mean ± SD/*n* %		Median	
BMI (kg/m^2^)		25.0±3.6		24.0	27.1±3.7		27.0	***.028***	^m^
Smoking	(−)	13	48.1%		21	84.0%		***.007***	^×^2^^
	(+)	14	51.9%		4	16.0%			
Comorbidity	(−)	21	77.8%		12	48.0%		***.026***	^×^2^^
	(+)	6	22.2%		13	52.0%			
HbA1c		6.3±1.2		6.0	6.4±1.0		6.0	.387	^m^
Duration of erectile dysfunction (years)		3.3±1.9		3.0	4.0±2.5		4.0	.369	^m^
***IEFF-5 score***									
Pre-ESWT		12.7±2.4		12.0	13.3±2.9		11.0	.886	^m^
Post-ESWT—1st month		21.9±2.8		22.0	17.6±3.9		18.0	***.000***	^m^
Post-ESWT—3rd month		22.1±2.5		23.0	19.0±3.6		19.0	***.001***	^m^
Post-ESWT—6th month		21.9±2.3		22.0	18.2±4.1		18.0	***.000***	^m^
***EHS***									
Pre-ESWT		1.5±0.8		2.0	1.4±0.7		1.0	.432	^m^
Post-ESWT—1st month		2.7±0.6		3.0	2.6±0.6		3.0	.509	^m^
Post-ESWT—3rd month		2.9±0.6		3.0	2.7±0.6		3.0	.395	^m^
Post-ESWT—6th month		2.7±0.5		3.0	2.5±0.7		3.0	.404	^m^
Total testosterone level before ESWT		494.2±224.7		435.0	378.1±124.6		345.0	***.010***	^m^

Abbreviations: BMI, body mass index; EHS, erection hardness score; ESWT, extracorporeal shock wave therapy; IIEF, International Index of Erectile Function.

m: Mann–Whitney *U* test; X^2^: chi-square test. Bold italic values indicate statistical significance (*P*<0.05).

The baseline IIEF scores were comparable between the 2 age groups prior to ESWT (12.7 ± 2.4 vs 13.3 ± 2.9, *P* > .05). In the < 50 years group, post-ESWT IIEF scores at 1, 3, and 6 months were 21.9, 22.1, and 21.9, respectively. In the ≥ 50 years group, the corresponding scores were 13.3, 17.6, and 19, respectively. International Index of Erectile Function scores increased at 1 and 3 months and stabilized by month 6. No further increase in IIEF scores was observed after month 3. Pretreatment EHSs were also comparable between the groups (1.5 ± 0.8 vs 1.4 ± 0.7). In the < 50 years group, EHSs at 1, 3, and 6 months post-ESWT were 2.7, 2.9, and 2.7, respectively. Similar to the IIEF results, the increase in EHS plateaued after month 3. In the ≥ 50 years group, EHSs at 1, 3, and 6 months post-ESWT were 2.6, 2.7, and 2.5, respectively (Table 2).

**Figure 1:**
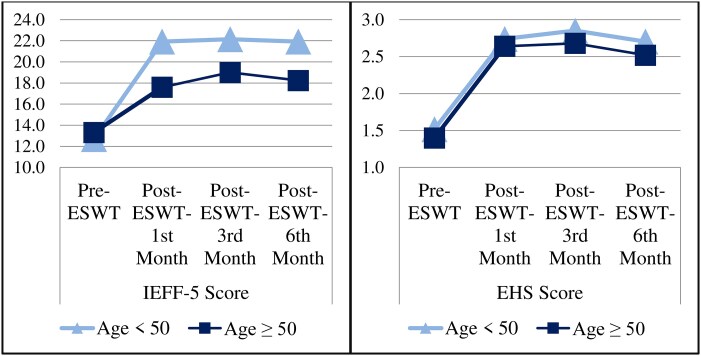
Change in IIEF scores and EHSs by age groups before and after ESWT. Abbreviations: EHS = erection hardness score; ESWT = extracorporeal shock wave therapy; IIEF = International Index of Erectile Function.

At 1, 3, and 6 months after ESWT, the IIEF-5 score in the group aged ≥ 50 years was significantly lower than that in the group aged < 50 years (*P* < .05). When comparing pretreatment and posttreatment values between the groups, no significant difference was detected in EHSs (*P* > .05). However, within each group, there was a significant increase in EHSs following ESWT (Figure 1).

Linear mixed model analysis demonstrated no significant difference in baseline IIEF-5 scores between the age groups (*P* = .474). A significant effect of time was observed (*P* < .001), indicating that IIEF-5 scores improved significantly after treatment in both groups. Furthermore, a significant age-by-time interaction was identified (all follow-up time points, *P* < .001), suggesting that the treatment response differed over time between the 2 age groups.

To evaluate the association between age and IIEF-5 scores while controlling for potential confounding factors, an ANCOVA was performed adjusting for smoking status, BMI, and the presence of comorbidities. After adjustment for smoking status, BMI, and comorbidities, no significant difference in baseline IIEF-5 scores was observed between the age groups (adjusted mean: 13.18 vs 13.04, ANCOVA, *P* = .869). At 1 month after the procedure, the adjusted IIEF-5 score was significantly higher in patients aged < 50 years compared with those aged ≥ 50 years (adjusted mean: 21.86 vs 17.91, ANCOVA, *P* < .001). This difference remained significant at the 3-month follow-up (adjusted mean: 22.02 vs 18.90, ANCOVA, *P* = .003). Similarly, at the 6-month follow-up, after adjustment for smoking status, BMI, and comorbidities, patients aged < 50 years continued to demonstrate significantly higher IIEF-5 scores than those aged ≥ 50 years (adjusted mean: 21.30 vs 17.82, ANCOVA, *P* = .002).

## Discussion

Low-intensity ESWT, which affects vascular and neurogenic mechanisms, has recently emerged as a promising noninvasive treatment modality for ED. The potential of LI-ESWT in ED treatment became apparent after early clinical and preclinical investigations in the 2010s.^4^ Animal studies have demonstrated that shock waves increase nitric oxide (NO) release in penile tissue and stimulate the expression of pro-angiogenic factors, such as vascular endothelial growth factor.^5^ In particular, rat models have shown that shock waves ameliorate cavernous nerve injury, promote neovascularization in penile tissue, and improve endothelial function.^6^ The proposed mechanism of LI-ESWT in the penis involves the induction of microtrauma at the microvascular level, which triggers a repair response characterized by neovascularization, endothelial regeneration, and neurogenic recovery.^7^

The efficacy of ESWT in ED may vary, particularly among patients with mild-to-moderate disease. Based on IIEF scores, greater improvements tend to be observed in the mild ED subgroup. Preservation of endothelial function and less irreversible cavernosal tissue damage in these patients might allow a more robust regenerative response to low-intensity shock waves.^8^^,^^9^ Therefore, we selected patients with mild and moderate ED for the present study, in whom ESWT is expected to be more effective.

In the present study, we compared the efficacy of ESWT between younger and older patients with mild-to-moderate ED and observed a more pronounced improvement in the younger patient group. In patients younger than 50 years, post-ESWT IIEF scores at 1, 3, and 6 months were 21.9, 22.1, and 21.9, respectively, whereas in patients aged 50 years and older, the corresponding scores were 13.3, 17.6, and 19, respectively (*P* < .005). Furthermore, the improvement observed at 1, 3, and 6 months after ESWT in younger patients remained consistent. However, in the older group, the improvement plateaued after month 3 and no significant improvement was observed thereafter. This finding suggested that ESWT may exert long-term benefits in younger patients.

Despite significant intergroup differences in post-ESWT IIEF scores, the improvement in EHSs remained similar. This inconsistency can be explained by the multidimensional nature of the IIEF in assessing various domains of sexual function, in contrast to the single-item EHS focusing solely on rigidity. Additionally, the inherent low sensitivity of the EHS might have masked potential differences between the groups.

There are several possible reasons why ESWT may be more effective in younger patients. First, younger individuals typically have better preserved vascular architecture, healthier endothelial function, and a greater capacity for neurogenic recovery, all of which may enhance treatment response. Previous studies have also shown that ESWT achieves the best results in the early stages of vascular ED.^3^^,^^10^ The findings of the present study were consistent with those of the previous studies and indicate that younger patients derive greater benefit from ESWT.

The lower efficacy of ESWT in older patients may be related to comorbidities commonly seen in this group, such as diabetes, hypertension, and cardiovascular disease. It is known that vascular degeneration in penile tissues increases and endothelial function deteriorates with advancing age.^7^ These factors might limit the angiogenic and tissue-regenerative effects of ESWT, thereby reducing treatment response. In the present study, we observed a reduced ESWT response in the older patient group compared with younger patients.

Previously, Ortac et al.^11^ reported that ESWT was more effective than placebo in treating young patients with mild erectile dysfunction. However, in their study, young patients were evaluated only within their own cohort. In contrast, in the present study, young patients were compared directly with older patients, with ESWT being more effective and yielding better long-term outcomes in younger patients. Another study reported that ESWT was effective for priapism-related ED in young patients, with higher IIEF scores at 3-month follow-up.^12^ In a retrospective study by Yao et al.,^13^ a combination of ESWT and a PDE5 inhibitor was given to young–middle-aged and older patients, and the young–middle-aged group demonstrated a better treatment response. However, in their study, it could not be distinguished whether the observed effect was attributable to ESWT or PDE5 inhibitors. The present study only included patients who received ESWT alone, and we showed that ESWT alone yields effective and durable results in the younger patient group. The success of ESWT as a monotherapy might obviate the need for adjunctive treatments, such as concomitant pharmacotherapy. Nevertheless, large-scale studies that include combined treatment arms are required to validate these findings.

This study has several limitations. First, the limited sample size restricts the generalizability of the results. Second, this study did not include a placebo-controlled group. Previous studies have demonstrated that placebo effects can play a significant role in ED treatment.^9^ While diabetes prevalence and HbA1c values were comparable across both cohorts, the inherently higher rate of comorbidities in patients aged over 50 remains a limitation, consistent with the known correlation between aging and multimorbidity. Larger, randomized, placebo-controlled trials are needed in the future to determine the efficacy of ESWT more comprehensively across different age groups.

## Conclusion

The findings indicated that ESWT is more effective in younger patients. Thus, age should be taken into account when planning ED treatment. However, long-term follow-up studies and large randomized controlled trials are warranted to validate these findings.

## Data Availability

The data that support the findings of this study are available from the corresponding author upon reasonable request.
